# Treatment of Pneumococcal Infection by Using Engineered Human C-Reactive Protein in a Mouse Model

**DOI:** 10.3389/fimmu.2020.586669

**Published:** 2020-10-07

**Authors:** Donald N. Ngwa, Sanjay K. Singh, Toh B. Gang, Alok Agrawal

**Affiliations:** Department of Biomedical Sciences, James H. Quillen College of Medicine, East Tennessee State University, Johnson City, TN, United States

**Keywords:** C-reactive protein, complement, factor H, pneumococcal infection, *Streptococcus pneumoniae*

## Abstract

C-reactive protein (CRP) binds to several species of bacterial pathogens including *Streptococcus pneumoniae*. Experiments in mice have revealed that one of the functions of CRP is to protect against pneumococcal infection by binding to pneumococci and activating the complement system. For protection, however, CRP must be injected into mice within a few hours of administering pneumococci, that is, CRP is protective against early-stage infection but not against late-stage infection. It is assumed that CRP cannot protect if pneumococci got time to recruit complement inhibitor factor H on their surface to become complement attack-resistant. Since the conformation of CRP is altered under inflammatory conditions and altered CRP binds to immobilized factor H also, we hypothesized that in order to protect against late-stage infection, CRP needed to change its structure and that was not happening in mice. Accordingly, we engineered CRP molecules (E-CRP) which bind to factor H on pneumococci but do not bind to factor H on any host cell in the blood. We found that E-CRP, in cooperation with wild-type CRP, was protective regardless of the timing of administering E-CRP into mice. We conclude that CRP acts via two different conformations to execute its anti-pneumococcal function and a model for the mechanism of action of CRP is proposed. These results suggest that pre-modified CRP, such as E-CRP, is therapeutically beneficial to decrease bacteremia in pneumococcal infection. Our findings may also have implications for infections with antibiotic-resistant pneumococcal strains and for infections with other bacterial species that use host proteins to evade complement-mediated killing.

## Introduction

C-reactive protein (CRP), whose blood level increases in inflammatory states in humans, binds to several species of bacterial pathogens, including *Streptococcus pneumoniae* (1–9). CRP is also a component of the acute phase response and a critical host defense molecule of the innate immune system against pneumococcal infection (10, 11). CRP binds to pneumococci by recognizing the phosphocholine (PCh) molecules present on the pneumococcal cell wall C-polysaccharide (PnC) (12). CRP is made of five identical subunits arranged as a cyclic pentamer (13). Each subunit has a PCh-binding site consisting of Phe^66^, Thr^76^ and Glu^81^ (13–18). Through the PCh-binding site, CRP can also interact with phosphoethanolamine (PEt) (17, 18). The binding of CRP to PCh and PEt requires two Ca^2+^ ions (13). Once complexed with a PCh-bearing ligand, human CRP activates the complement system in both human and murine sera (3, 19–21). The amino acid residues Tyr^40^ and Glu^42^ investigated in the current study are part of the intersubunit contact region of the CRP pentamer (13–15).

In murine models of pneumococcal infection, human CRP has been shown to be protective against lethality; however, the molecular mechanism of anti-pneumococcal action of CRP remains undefined (18, 21–31). Interestingly, CRP is protective against pneumococcal infection only when injected 6 h before to 2 h after administering pneumococci into mice, that is, CRP is protective against early-stage infection but not against late-stage infection (24). This 36-year old observation provided us with an experimental strategy to define the mechanism of anti-pneumococcal functions of CRP in mice. CRP is protective against early-stage infection because of the ability of CRP-pneumococci complexes to activate the complement system (20, 31). The reason why CRP is not protective against late-stage pneumococcal infection is not known. It is assumed that after a few hours of initial infection, pneumococci recruit the complement inhibitory protein factor H on their surface to become complement attack-resistant (32–36).

It has been shown that the pentameric conformation of CRP is altered in a variety of experimental conditions mimicking an inflammatory milieu and, in its alternate pentameric conformation, CRP binds to immobilized complement inhibitor factor H also (37–47). We hypothesize that in order to protect mice against late-stage infection, a structural change in CRP is needed, followed by the interaction between structurally altered CRP and recruited factor H on the pneumococcal surface, and that was not happening in mice. We further hypothesize that an exogenously constructed, irreversible and stable CRP mutant capable of binding to factor H should be able to protect mice against late-stage infection; such a CRP molecule when administered into mice would bind to factor H on pneumococci *in vivo* and mask the complement inhibitory activity of factor H (48). Wild-type (WT) CRP should then be able to protect mice against otherwise complement-resistant pneumococci by activating the complement system if a CRP molecule which can cover factor H on pneumococci is present along with WT CRP.

In this study, to test our hypotheses, we engineered CRP (E-CRP) by site-directed mutagenesis and produced two types of E-CRP: One, E42Q/F66A/T76Y/E81A, that binds to immobilized factor H but does not bind to PCh (E-CRP-1) and another, Y40F/E42Q, that binds to both immobilized factor H and to PCh (E-CRP-2). We found that both E-CRP-1 and E-CRP-2 were protective against both early-stage and late-stage infection in a murine model of pneumococcal infection. These findings indicate that CRP functions in two different structural conformations to fully protect against pneumococcal infection.

## Materials and Methods

### Construction of Mutant CRP cDNAs

The template for construction of the CRP quadruple mutant E42Q/F66A/T76Y/E81A (E-CRP-1) was a cDNA for the CRP triple mutant F66A/T76Y/E81A cDNA (substitution of Phe^66^ with Ala, Thr^76^ with Tyr, and Glu^81^ with Ala). Mutagenic oligonucleotides, 5′-C CAC TTC TAC ACG ***CAA*** CTG TCC TCG ACC-3′ and 5′-GGT CGA GGA CAG ***TTG*** CGT GTA GAA GTG G-3′, to substitute Glu^42^ with Gln (codons shown in bold and italicized letters), were designed according to the sequence of the template cDNA and obtained from Integrated DNA Technologies. Mutagenesis was conducted using the QuickChange site-directed mutagenesis kit (Stratagene). Mutations were verified by nucleotide sequencing, utilizing the services of the Molecular Biology Core Facility of the university (Supplementary Figure 1). The construction of cDNAs for CRP mutants E42Q, F66A/T76Y/E81A and Y40F/E42Q (E-CRP-2) has been reported earlier (15–18).

### Expression and Purification of CRP

CRP mutants were expressed in CHO cells using the ExpiCHO Expression System (Thermo Fisher Scientific) as described previously (31). Purification of E-CRP-1 from culture supernatants involved Ca^2+^-dependent affinity chromatography on a PEt-conjugated Sepharose column, followed by ion-exchange chromatography on a MonoQ column, and gel filtration on a Superose12 column, as reported previously for the CRP triple mutant F66A/T76Y/E81A (18). PEt-conjugated Sepharose was prepared as described previously (18). Briefly, CHO cell culture media was diluted (1:1) in 0.1 M borate buffer saline, pH 8.3, containing 3 mM CaCl_2_, and passed through the PEt-sepharose column. After collecting the flow-through and washing the column with the same buffer, bound E-CRP-1 was eluted with 0.1 M borate buffer saline, pH 8.3, containing 5 mM EDTA. E-CRP-1 was then subjected to ion-exchange chromatography and bound E-CRP-1 was eluted with an NaCl gradient. E-CRP-1 containing fractions were pooled, concentrated, and further purified by gel filtration. The gel filtration column was equilibrated and eluted with TBS (10 mM Tris–HCl, 150 mM NaCl, pH 7.2) containing 5 mM EDTA. Eluted E-CRP-1 was immediately dialyzed against TBS containing 2 mM CaCl_2_, stored at 4°C, and was used within a week. WT CRP and all other CRP mutants including E-CRP-2 were purified as described previously (49). The purity of CRP preparations was confirmed by denaturing 4–20% SDS-PAGE under reducing conditions.

For *in vivo* experiments, purified CRP was treated with Detoxi-Gel Endotoxin Removing Gel (Thermo Fisher Scientific) according to manufacturer’s instructions. The concentration of endotoxin in CRP preparations was determined by using the Limulus Amebocyte Lysate kit QCL-1000 (Lonza).

### Determination of Pentameric Structure of CRP

The pentameric structure of E-CRP was confirmed by employing gel filtration and denaturing SDS-PAGE. The gel filtration column was equilibrated with TBS containing 5 mM EDTA. E-CRP was injected into the column and eluted with TBS containing 5 mM EDTA at a flow rate of 0.3 ml/min. Fractions (60 fractions, 250 μl each) were collected and absorbance at 280 nm measured to locate the elution volume of E-CRP. Gel filtration of WT CRP was carried out on the same column to determine the elution volume of pentameric CRP.

### Pneumococci (Pn-Broth)

*Streptococcus pneumoniae* type 3, strain WU2, were made virulent by sequential i.v. passages in mice, and were stored in 1 ml aliquots at −80°C in Todd-Hewitt broth containing 0.5% yeast extract and 10% glycerol, as described previously (30). For each experiment, a separate 1 ml aliquot of pneumococci was thawed. Pneumococci were then grown in 50 ml Todd-Hewitt broth containing 0.5% yeast extract and incubated at 37°C with shaking at 125 rpm for 3 h (mid-log phase culture). The culture was centrifuged at 7,500 rpm for 15 min. The bacterial pellet was washed and resuspended in 10 ml normal saline and adjusted the volume until *A*_600_ was 0.29 to give a concentration of 3.5 × 10^8^ cfu/ml (*A*_600_ = 1.00 = 1.2 × 10^9^ cfu/ml). This preparation of pneumococci cultured in broth was called as Pn-broth. The concentration, purity, and viability of pneumococci were confirmed by plating on sheep blood agar plates.

### PCh-Binding, PEt-Binding, and Pneumococcus-Binding Assays

Binding activity of CRP for PCh was evaluated by using pneumococcal C-polysaccharide (PnC, from Statens Serum Institut) as the ligand, as described previously (29). Briefly, microtiter wells were coated with PnC in 100 μl TBS, overnight at 4°C. The unreacted sites were blocked with TBS containing 0.5% gelatin for 1 h at room temperature. CRP, diluted in TBS containing 2 mM CaCl_2_, 0.1% gelatin and 0.02% Tween 20 (TBS-Ca), was then added in duplicate wells and incubated for 2 h at 37°C. After washing the wells with TBS-Ca, bound CRP was detected by using anti-CRP monoclonal antibody HD2.4 diluted in TBS-Ca. HRP-conjugated goat anti-mouse IgG diluted in TBS-Ca was used as the secondary antibody. Color was developed using ABTS substrate and the OD was read at 405 nm in a plate reader. Binding activity of CRP for PEt was evaluated by using biotinylated-PEt as the ligand, exactly as described previously (18).

Binding activity of CRP for whole pneumococci (broth-grown or isolated from infected mice) was evaluated as described previously (18). Briefly, microtiter wells were coated with 10^7^ cfu of pneumococci overnight at 4°C. The unreacted sites in the wells were blocked with TBS containing 0.5% gelatin. CRP, diluted in TBS-Ca, was then added to the wells for 2 h at 37°C. After washing the wells with TBS-Ca, bound CRP was detected by using rabbit polyclonal anti-human CRP antibody. HRP-conjugated donkey anti-rabbit IgG was used as the secondary antibody. Color was developed using ABTS substrate and the OD was read at 405 nm in a plate reader. Binding activity of CRP for whole pneumococci isolated from infected mice was evaluated both in the presence and absence of Ca^2+^.

### Isolation of Pneumococci (Pn-Mice) From Infected Mice

Mice were injected i.v. with 3.5 × 10^7^ cfu of Pn-broth. After 40 h, blood was collected by cardiac puncture, in tubes containing 10% EDTA (1% v/v of blood). Blood was diluted with an equal volume of normal saline and centrifuged at 2,200 rpm for 2 min. The supernatant was recovered. The bacterial pellet was washed four times with normal saline, centrifuged at 2,200 rpm for 2 min after each wash, and continued to recover the supernatant. All recovered supernatants were then pooled and centrifuged at 11,000 rpm for 5 min. This time the supernatant was discarded, and the pellet was resuspended in normal saline for immediate use or resuspended in Todd-Hewitt broth containing 0.5% yeast extract and 10% glycerol for storage at −80°C. This preparation of pneumococci isolated from infected mice was called as Pn-mice. The concentration, purity, and viability of pneumococci were confirmed by plating on sheep blood agar plates.

### Detection of Factor H on the Surface of Pn-Mice

Microtiter wells were coated with Pn-mice in TBS (10^7^ cfu) overnight at 4°C. The unreacted sites in the wells were blocked with TBS containing 0.5% gelatin for 45 min at room temperature. Murine factor H present on the surface of Pn-mice was detected by using sheep polyclonal anti-mouse factor H antibody (R&D, AF4999) diluted in TBS-Ca. HRP-conjugated rabbit anti-sheep IgG (Thermo Fisher Scientific), in TBS-Ca, was used as the secondary antibody. Color was developed and the OD_405_ read in a microtiter plate reader.

### Factor H-Binding Assay

The binding activity of CRP for factor H was evaluated by using both human factor H (Complement Technology) and murine factor H (R&D), as described previously (46). Briefly, microtiter wells were coated with 2 μg/ml of factor H in TBS, overnight at 4°C. The unreacted sites in the wells were blocked with TBS containing 0.5% gelatin for 45 min at room temperature. CRP diluted in TBS-Ca (TBS containing 2 mM CaCl_2_, 0.1% gelatin and 0.02% Tween 20) was added in duplicate wells. After incubating the plates for 2 h at 37°C, the wells were washed with TBS-Ca. Polyclonal rabbit anti-human CRP antibody (1 μg/ml) (EMD Millipore Corp, 235752), diluted in TBS-Ca, was used to detect bound CRP. HRP-conjugated donkey anti-rabbit IgG (GE Healthcare), diluted in TBS-Ca, was used as the secondary antibody. Color was developed and the OD_405_ read in a microtiter plate reader.

### Clearance of E-CRP From Mouse Circulation

The clearance rate of E-CRP from the mouse blood was determined as described previously (18). Briefly, mice were injected i.v. with 100 μg of CRP in 100 μl TBS containing 2 mM CaCl_2_ through the tail vein. Four to five mice were used for each CRP species. After 8 h, blood was collected from the tip of the tail vein at four different time points up to 24 h. The concentration of CRP in the serum was measured by ELISA. The concentration of CRP in the serum at the first bleed was plotted as the 100% value.

### Repurification of E-CRP From E-CRP-Spiked Mouse Serum

Purified E-CRP (400 μg) was added to 2 ml C57BL/6 mouse serum (Innovative Research) and the final volume was made to 10 ml by adding 0.1 M borate buffered saline, pH 8.3, containing 3 mM CaCl_2_. The mixture was incubated for 30 min at 37°C. E-CRP was repurified by Ca^2+^-dependent affinity chromatography on PEt-Sepharose beads whose capacity to bind E-CRP was >400 μg. After collecting the flow-through and washing the column with the same buffer, bound E-CRP was eluted with 0.1 M borate buffered saline, pH 8.3, containing 5 mM EDTA. To control the experiment, mouse serum alone (2 ml), without spiking with E-CRP, was used. The EDTA eluates were subjected to SDS-PAGE. The concentration of CRP in the EDTA eluates was measured by ELISA to calculate percent recovery.

### Mice

Male C57BL/6J mice (Jackson Laboratories) were brought up and maintained according to protocols approved by the University Committee on Animal Care. Mice were 8–10 weeks old when used in experiments.

### Mouse Protection Experiments

Separate mouse protection experiments were performed using two different preparations of purified WT CRP, E-CRP-1 and E-CRP-2. The endotoxin content in 25 μg all CRP preparations was <1.5 endotoxin units. Mice were first injected i.v. with 3.5 × 10^7^ cfu (based on *A*_600_) of pneumococci in 100 μl normal saline. The actual number of pneumococci injected, based on the plating results obtained on the next day, was 3.53 ± 0.21 × 10^7^ cfu. In the first set of experiments, mice were injected i.v. with either WT CRP, E-CRP-1 or E-CRP-2, 30 min after the administration of pneumococci. In the second set of experiments, mice were injected i.v. with either WT CRP, E-CRP-1, combination of WT CRP and E-CRP-1 (WT CRP first and, an hour later, E-CRP-1) or E-CRP-2, 12 h after the administration of pneumococci. In the third set of experiments, mice were injected i.v. with either WT CRP or E-CRP-1, four times (6, 12, 24 and 48 h) after the administration of pneumococci. CRP (25 μg) was injected in 100 μl TBS containing 2 mM CaCl_2_. The dose of 25 μg of CRP with 3.5 × 10^7^ cfu bacteria was chosen because, under these conditions, the protection of mice with WT CRP injected 30 min apart from the administration of pneumococci was same as reported previously (30). Survival of mice was recorded three times per day for 7 days. To determine bacteremia (cfu/ml) in the surviving mice, blood was collected daily for 5 days from the tip of the tail vein, diluted in normal saline, and plated on sheep blood agar for colony counting. The bacteremia value for dead mice was recorded as 10^9^ cfu/ml because mice died when the bacteremia exceeded 10^8^ cfu/ml.

### Statistical Analysis

All experiments were performed three times unless otherwise mentioned and comparable results were obtained each time. Results of a representative experiment are shown in the figures where the raw data (*A*_280_ or OD_405_) were used to plot the curves. Survival curves were generated using the GraphPad Prism 4 software. To determine *p*-values for the differences in the survival curves among various groups, the survival curves were compared using the software’s Logrank (Mantel-Cox) test. The scatter plots of the bacteremia data and the median bacteremia value for each group were generated using the GraphPad Prism 4 software. Bacteremia values of 0–100 were plotted as 100 and bacteremia values of >10^8^ were plotted as 10^9^. To determine *p*-values for the differences in bacteremia among various groups at each time point, scatter plots were compared using the software’s Mann-Whitney test. The software’s Mann-Whitney test included all the dots in the scatter plots and not the median values for each time point.

## Results

### E-CRP-1 and E-CRP-2 Have the Desired Ligand-Binding Properties

**FIGURE 1:**
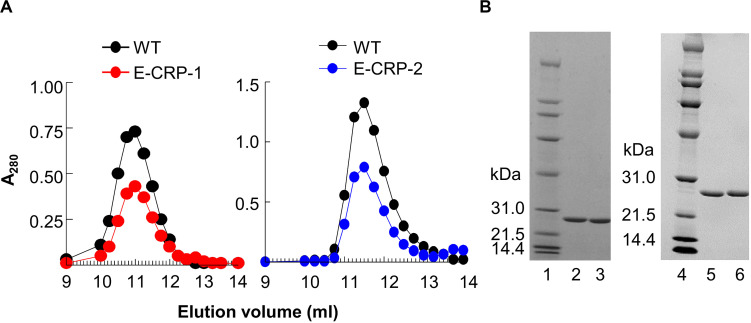
E-CRP-1 and E-CRP-2 are pentameric. **(A)** Elution profiles of E-CRP-1 (left panel) and E-CRP-2 (right panel) from the gel filtration column. **(B)** SDS-PAGE of WT CRP (lanes 2, 5), E-CRP-1 (lane 3) and E-CRP-2 (lane 6). A representative of three experiments is shown for each panel.

The elution profiles of WT CRP, E-CRP-1 and E-CRP-2 from the gel filtration column were almost overlapping. Like WT CRP, both E-CRP-1 and E-CRP-2 eluted at 11 ml (Figure 1A). SDS-PAGE of purified proteins showed a single band and the molecular weight of the subunits of both E-CRP-1 and E-CRP-2 was same as WT CRP (Figure 1B). Thus, E-CRP-1 and E-CRP-2 were pentameric.

**FIGURE 2:**
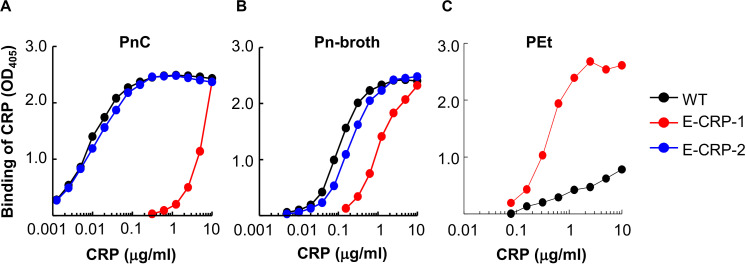
Binding of CRP to PCh. **(A)** Binding of WT CRP, E-CRP-1 and E-CRP-2 to PnC immobilized on microtiter wells. **(B)** Binding of WT CRP, E-CRP-1 and E-CRP-2 to broth-cultured pneumococci (Pn-broth) immobilized on microtiter wells. **(C)** Binding of WT CRP and E-CRP-1 to a PEt-ligand immobilized on microtiter wells. A representative of three experiments is shown for each panel.

The PCh-binding ability of E-CRP-1 and E-CRP-2 was assessed by using two different PCh-containing ligands: PnC (Figure 2A) and Pn-broth (Figure 2B). Both, WT CRP and E-CRP-2 bound to both PCh-ligands in a CRP concentration-dependent manner. The binding of E-CRP-2 to PCh-ligands was comparable to that of WT CRP. However, as shown, for equivalent binding (OD_405_) of WT CRP and E-CRP-1 to either PnC or Pn-broth, ∼100-times more of E-CRP-1 was required compared to WT CRP, indicating that the PCh-binding ability of E-CRP-1 was ∼99% less than that of WT CRP. Thus, E-CRP-1 and E-CRP-2 had the desired PCh-binding activity.

Since E-CRP-1 lost its PCh-binding property, the PCh-affinity chromatography method could not be used to purify E-CRP-1. Therefore, the PEt-binding activity of E-CRP-1 was tested (Figure 2C). E-CRP-1 bound to PEt more efficiently than WT CRP. The avid binding of E-CRP-1 to PEt facilitated the purification of E-CRP-1 by affinity chromatography using a PEt-Sepharose column.

**FIGURE 3:**
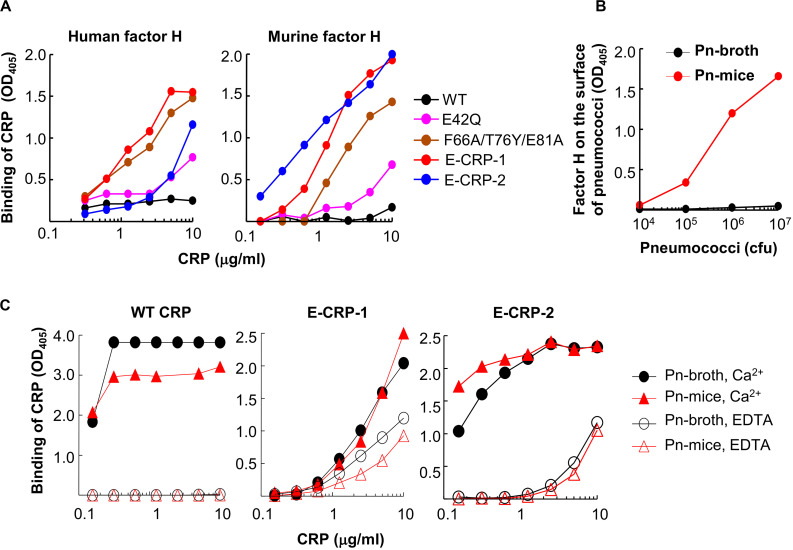
Binding of CRP to factor H. **(A)** Binding of WT CRP, E-CRP-1, E-CRP-2 and other CRP mutants to purified human factor H (left panel) and murine factor H (right panel) immobilized on microtiter wells. **(B)** Presence of murine factor H on pneumococci isolated from the blood of infected mice (Pn-mice). **(C)** Binding of WT CRP, E-CRP-1 and E-CRP-2 to Pn-broth and Pn-mice in the presence and absence of Ca^2+^. A representative of three experiments is shown for each panel.

In factor H-binding assays, unlike WT CRP, both E-CRP-1 and E-CRP-2 bound readily to purified human and murine factor H immobilized on microtiter wells (Figure 3A). Thus, both E-CRP-1 and E-CRP-2 had the desired factor H-binding activity. Surprisingly, triple mutant CRP, which was not investigated before for factor H binding (18), also bound to factor H (50). If it was known earlier that triple mutant CRP, without the addition of E42Q mutation, would bind to factor H, there would have been no need to generate quadruple mutant CRP. However, since the expression of quadruple mutant CRP was better than triple mutant CRP, we proceeded with quadruple mutant CRP (E-CRP-1) to test the hypothesis.

Next, we tested whether E-CRP-1 and E-CRP-2 bind to factor H on pneumococci which have recruited factor H on their surface. For this purpose, Pn-mice were isolated from infected mice. We first tested the presence of murine factor H on pneumococci. As shown (Figure 3B), factor H was present on Pn-mice but not on Pn-broth. Next, we determined binding of E-CRP-1 and E-CRP-2 to factor H-coated Pn-mice. As shown (Figure 3C), WT CRP bound to both Pn-broth and Pn-mice but only in the presence of Ca^2+^, suggesting that the binding of WT CRP to Pn-mice was through PCh. In contrast, E-CRP-1 and E-CRP-2 bound to Pn-mice in the absence of Ca^2+^ also, suggesting that E-CRP-1 and E-CRP-2 bound to a molecule other than PCh, and that molecule could be factor H recruited by pneumococci *in vivo*. However, interestingly, both E-CRP-1 and E-CRP-2 also bound to Pn-broth in EDTA, suggesting that E-CRP-1 and E-CRP-2 were capable of recognizing and binding to a pneumococcus’ own surface protein. Thus, E-CRP-1 and E-CRP-2, but not WT CRP, interacted with pneumococci in a Ca^2+^-independent and therefore PCh-independent manner.

Combined data indicated that both E-CRP-1 and E-CRP-2 were pentameric, their overall structure was similar to WT CRP, and both E-CRP-1 and E-CRP-2 had the desired ligand-binding properties to test our hypothesis.

### Both E-CRP-1 and E-CRP-2 Are Suitable for *in vivo* Use

**FIGURE 4:**
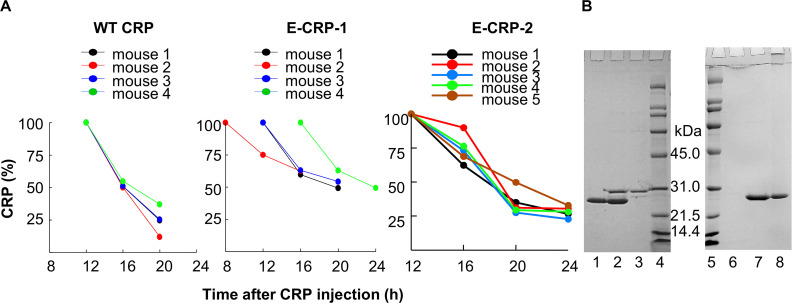
E-CRP-1 and E-CRP-2 are suitable for *in vivo* use. **(A)** Clearance of WT CRP (left panel), E-CRP-1 (middle panel) and E-CRP-2 (right panel) from mouse circulation. **(B)** Repurification of E-CRP-1 and E-CRP-2 from purified E-CRP-1-spiked and E-CRP-2-spiked mouse sera, respectively. SDS-PAGE of repurified E-CRP-1 and E-CRP-2 is shown. Lane 1, purified E-CRP-1; Lane 2, EDTA eluate from the PEt-affinity chromatography column through which mouse serum containing E-CRP-1 was passed in the presence of Ca^2+^; Lane 3, EDTA eluate from the PEt-column through which mouse serum alone was passed. Lane 6, EDTA eluate from the PCh-affinity chromatography through which mouse serum alone was passed; Lane 7, EDTA eluate from the PCh-column through which mouse serum containing E-CRP-2 was passed in the presence of Ca^2+^; Lane 8, purified E-CRP-2.

We determined the *T*_1__/__2_ of CRP from mouse circulation. Based on the data obtained from four to five mice (Figure 4A), the average *T*_1__/__2_ of WT CRP, E-CRP-1 and E-CRP-2 were 4.9, 8.0 and 7.5 h, respectively. Thus, the clearance of E-CRP-1 and E-CRP-2 was not markedly faster than that of WT CRP. In another approach to confirm that E-CRP-1 and E-CRP-2 were free in the mouse serum, we performed an experiment where E-CRP could be repurified from E-CRP-spiked mouse serum (Figure 4B). As shown (left panel), E-CRP-1 present in the mouse serum bound to PEt in a Ca^2+^-dependent manner and could be eluted with EDTA (lane 2). The recovery of E-CRP-1 was 96%. Besides CRP, no additional protein bands were found when compared with the non-specific bands seen with the serum alone control (compare lanes 2 and 3). Similar results were seen with E-CRP-2 (right panel). Thus, both E-CRP-1 and E-CRP-2 stayed free in the mouse serum, were not sequestered by any other serum protein, and the mutations did not confer instability to E-CRP-1 and E-CRP-2 *in vivo*.

To test the possibility that E-CRP-1 and E-CRP-2 may be sequestered by cells in the mouse blood, E-CRP-spiked mouse blood was centrifuged at 8000 rpm for 5 min and the serum recovered. The amount of E-CRP in the recovered serum, as determined by ELISA, was the same as the amount of E-CRP mixed with the blood. These data showed that both E-CRP-1 and E-CRP-2 were suitable for use in mouse models of infection to test the hypothesis.

### Role of Endogenous Murine CRP in the Animal Model

**FIGURE 5:**
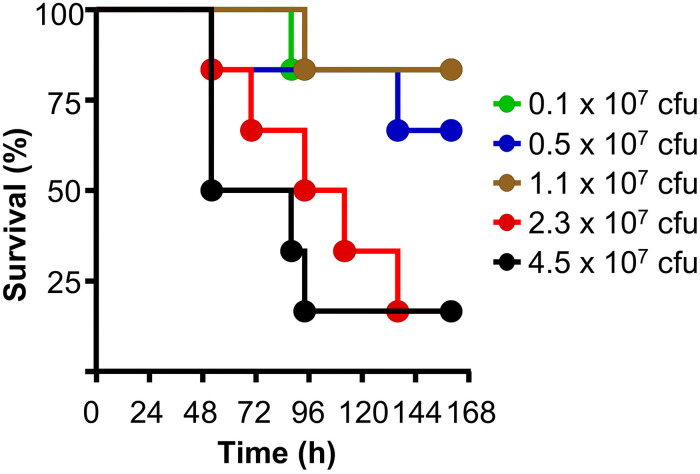
The animal model of pneumococcal infection. Survival curves of mice infected with different doses of pneumococci. The data are combined from two separate experiments with six to eight mice for each dose of pneumococci in each experiment.

As shown in Figure 5, mice were protected, without administering human CRP, when up to 10^7^ cfu of pneumococci were injected into mice, suggesting that endogenous murine WT CRP was sufficient to protect mice from lethality when bacteremia was relatively lower. Endogenous murine CRP was not enough to protect mice from lethality when >10^7^ cfu of pneumococci were injected into mice. These data also suggest that if mice are administered with, for example, 3.5 × 10^7^ cfu bacteria in the protection experiments, endogenous murine CRP can participate in protecting mice from lethality once bacteremia is lowered to <10^7^ cfu of pneumococci in the animal model employed in this study.

### E-CRP-1 Protects Mice Against Late-Stage Infection

**FIGURE 6:**
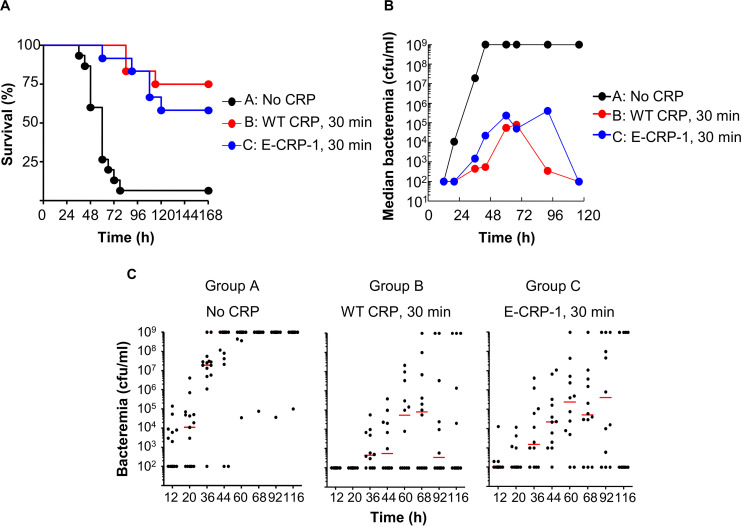
Like WT CRP, E-CRP-1 is also protective against early-stage infection. The data are combined from two separate experiments with six to eight mice in each group in each experiment. **(A)** Survival curves. Pneumococci and CRP were injected 30 min apart. The *p*-values for the differences in the survival curves between groups A B, A C, and B C were <0.001, <0.001 and 0.43, respectively. **(B)** Bacteremia. Blood was collected from each surviving mouse shown in **A**. The median bacteremia values are plotted. For 36-116 h, the *p*-values for the differences between groups A B and A C were <0.001. The *p*-value for the difference between groups B C was >0.05 at all time points. **(C)** Scatter plots of the bacteremia data shown in **B**. The horizontal line in each group of mice represents median bacteremia.

**FIGURE 7:**
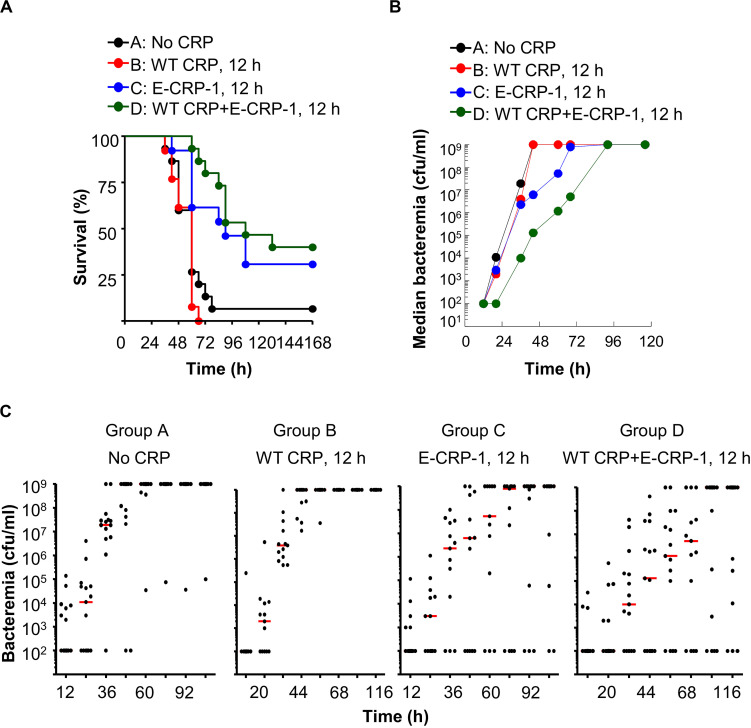
Unlike WT CRP, E-CRP-1 is protective against late-stage infection. The data are combined from two separate experiments with six to eight mice in each group in each experiment. **(A)** Survival curves. Pneumococci were injected first; CRP was injected 12 h later. The *p*-values for the differences in the survival curves between groups A B, A C, A D, B C, B D and C D were 0.28, <0.01, <0.001, <0.001, <0.001 and 0.31, respectively. **(B)** Bacteremia. Blood was collected from each surviving mouse shown in **A**. The median bacteremia values are plotted. The *p*-values for the differences between groups A B and C D were >0.05 at all time points. For 44–92 h, the *p*-values for the differences between groups B C and B D were <0.01. **(C)** Scatter plots of the bacteremia data shown in **B**. The horizontal line in each group of mice represents median bacteremia.

**FIGURE 8:**
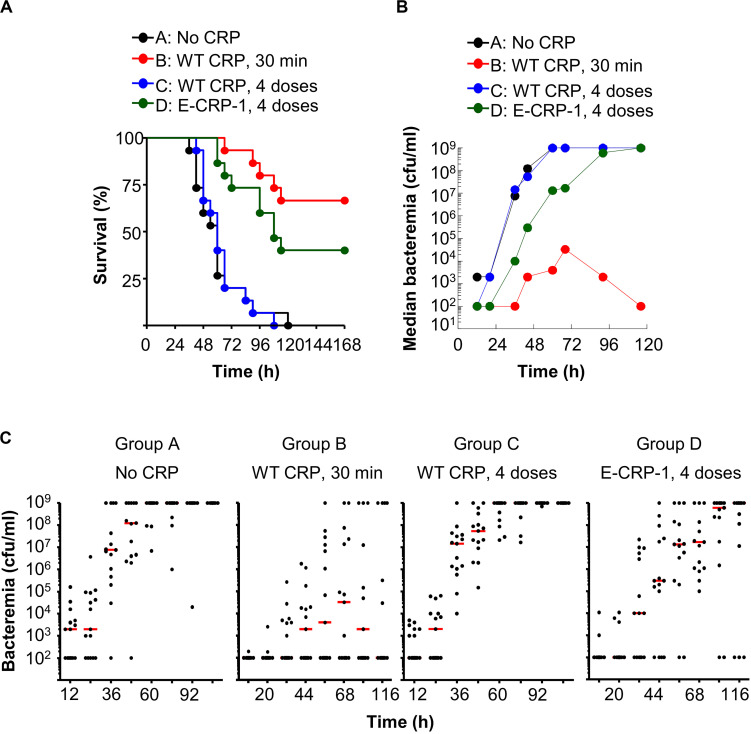
One injection of E-CRP-1 was sufficient to protect against late-stage infection. The data are combined from two separate experiments with six to eight mice in each group in each experiment. **(A)** Survival curves. CRP was injected four times: 6, 12, 24 and 48 h after injecting pneumococci. The *p*-values for the differences in the survival curves between groups A B, A D, B C and C D were <0.001. **(B)** Bacteremia. Blood was collected from each surviving mouse shown in **A**. The median bacteremia values are plotted. The *p*-value for the difference between groups A C was >0.05 at all-time points. For 36–116 h, the *p*-value for the difference between groups B C was <0.001. For 44–116 h, the *p*-values for the differences between groups B D and C D were <0.05. For 44-116 h, the *p*-value for the difference between groups A D was <0.01. **(C)** Scatter plots of the bacteremia data shown in **B**. The horizontal line in each group of mice represents median bacteremia.

All the data presented in this section show the combined results of two separate protection experiments using six to eight mice in each group in each experiment. Some protection experiments shown in Figures 6–8 were performed together.

Figure 6A shows the results of experiments in which WT CRP and E-CRP-1 were injected into mice within 30 min of administering pneumococci. The median survival time (MST, the time taken for the death of 50% of mice) for mice injected with bacteria alone (group A) was 60 h. The MST for mice injected with bacteria and either WT CRP (group B) or E-CRP-1 (group C) could not be calculated because >50% of mice survived. WT CRP and E-CRP-1 were not significantly different in protecting mice from lethality. Increase in survival was due to decrease in bacteremia (Figures 6B,C). By 44 h, in group A, median bacteremia increased dramatically, and mice died once bacteremia reached 10^9^ cfu/ml; however, in groups B and C, median bacteremia reached only ∼10^5^ cfu/ml and then decreased dramatically afterward. There was >99% reduction in bacteremia in both WT CRP-treated and E-CRP-1-treated mice. Since E-CRP-1 does not bind to PCh, and hence cannot activate the complement system, these results indicated that the increased resistance to infection in E-CRP-1-treated mice was due to combined actions of E-CRP-1 and endogenous mouse WT CRP. Most likely, E-CRP-1 bound to a protein ligand present on the pneumococcal surface and, once bacteremia was already lower, endogenous mouse WT CRP bound to PCh to activate the complement system to reduce bacteremia further. Since the dose of injected E-CRP-1 was same as that of WT CRP, it is unlikely that the protection depended upon the residual PCh-binding activity of E-CRP-1.

Next, we injected E-CRP-1 into mice 12 h after administering pneumococci, a time point for CRP injection when WT CRP does not confer protection (Figure 7A). A gap of 12 h is clinically significant because all strategies for a sepsis drug have so far failed in human clinical trials (51, 52). We included WT CRP, 30 min regimen, in all experiments to ensure that the animal model was comparable from experiment to experiment. The MST for mice injected with either bacteria alone (group A) or with bacteria and WT CRP (group B) was 60 h. In contrast, the MST for mice injected with bacteria and E-CRP-1 (group C) was 90 h and the MST for mice injected with bacteria and both WT CRP and E-CRP-1 (group D) was 108 h. In the WT CRP-treated group, all mice died by 66 h. However, in the E-CRP-1-treated groups, it took 4 days until 60–70% mice died, and 30–40% mice survived up to 7 days. As reported previously (24, 29), WT CRP was not protective. These data again suggested that endogenous mouse WT CRP participated and that is why E-CRP-1 alone was not different from the combination of E-CRP-1 and WT CRP in protecting mice from lethality. In mice receiving E-CRP-1 (groups C and D), median bacteremia was reduced by ∼99% as early as 44 h and lower bacteremia was maintained for up to 92 h (Figures 7B,C).

Next, we injected CRP into mice four times, at 6, 12, 24 and 48 h, after administering pneumococci, to determine whether multiple injections of E-CRP-1 were better than a single injection at 12 h (Figure 8A). The MST for mice injected with either bacteria alone (group A) or with bacteria and four doses of WT CRP (group C) was 60 h. Like a single dose of WT CRP at 12 h, multiple doses of WT CRP were also not protective. In contrast, the MST for mice injected with bacteria and multiple doses of E-CRP-1 (group D) was 108 h. In E-CRP-1-treated mice, median bacteremia was reduced by ∼99% as early as 36 h and the reduction lasted for up to 72 h. There was ∼48 h gain over WT CRP for bacteremia to reach the deadly levels (Figures 8B,C).

The protective ability of E-CRP-1 when injected at 30 min, 12 h or at multiple time points were compared (Supplementary Figure 2). The injection of E-CRP-1 at 12 h was found to be as effective as it was when administered within 30 min. Based on the statistical analyses of the survival curves and of the scatter plots for bacteremia (Figures 6–8), no significant difference was found between 30 min and 12 h regimens in either the survival of or bacteremia in both groups of mice (Supplementary Figures 2A,B). Likewise, four injections of E-CRP-1 and one injection of E-CRP-1 were equally effective in reducing bacteremia; there was no significant difference in either the survival of or bacteremia in these two groups of mice (Supplementary Figures 2C,D). In this study, mice were injected with E-CRP-1 four times within 48 h of administering pneumococci. It is possible that a different regimen for four injections of E-CRP-1 that improves its availability over the course of the infection, such as four injections spread over 4 days, would have shown results different from that used in this study and, therefore, be more protective than a single injection of E-CRP-1. Overall, the data indicate that E-CRP-1, unlike WT CRP, is protective against infection regardless of the time point of injecting E-CRP-1.

### E-CRP-2 Also Protects Mice Against Late-Stage Infection

**FIGURE 9:**
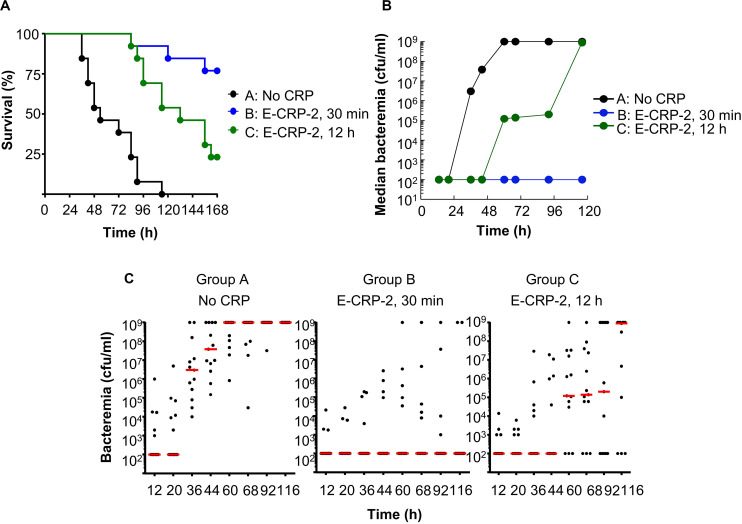
Like E-CRP-1, E-CRP-2 is also protective against late-stage infection. The data are combined from two separate experiments with six to eight mice in each group in each experiment. **(A)** Survival curves. CRP was injected first; pneumococci were injected either 30 min or 12 h later. The *p*-values for the differences in the survival curves between groups A C and A B were <0.001 and between groups B C was 0.01. **(B)** Bacteremia in each surviving mouse shown in **A**. The median values of bacteremia are plotted. For 36–116 h, the *p*-values for the differences between groups A B and A C were <0.001. The *p*-values for the differences between groups B C was >0.05 till 60 h and <0.05 after 60 h. **(C)** Scatter plots of the bacteremia data shown in **B**. The horizontal line in each group of mice represents median bacteremia.

The results of protection experiments with E-CRP-2 are shown in Figure 9. The MST for mice injected with bacteria alone (group A) was 54 h. The MST for mice injected with E-CRP-2, 12 h after administering pneumococci (group C), was extended to 132 h. The MST for mice injected with E-CRP-2, 30 min after administering pneumococci (group B), could not be calculated because >50% of mice survived, as expected (Figure 9A). There was >99% reduction in bacteremia even when E-CRP-2 was given to mice 12 h after administering pneumococci and the lower bacteremia stayed as such for >96 h (Figures 9B,C). Since E-CRP-2 binds to PCh, like WT CRP does, we do not know the involvement of mouse endogenous WT CRP in this case.

## Discussion

In this study, two CRP mutants, E-CRP-1 and E-CRP-2, were employed to investigate the mechanisms of anti-pneumococcal function of CRP in a mouse model of pneumococcal infection. E-CRP-1 binds to factor H but does not bind to PCh and E-CRP-2 binds to both factor H and PCh. Our major findings were: 1. CRP with mutations in either the intersubunit contact region or in the overlapping PCh-binding and Ca^2+^-binding sites binds to immobilized factor H. 2. Unlike WT CRP, both E-CRP-1 and E-CRP-2 were protective against late-stage infection. 3. Injecting WT CRP along with E-CRP-1 into mice did not enhance the protective ability of E-CRP-1. 4. Multiple injections of E-CRP-1 were not different from a single injection in protecting mice against infection. 5. Unlike WT CRP, both E-CRP-1 and E-CRP-2 bound to broth-grown pneumococci in the absence of Ca^2+^. Overall, these findings indicate that a conformationally altered form of CRP capable of binding to factor H is necessary for protection against late-stage pneumococcal infection.

We previously reported a triple mutant of CRP, F66A/T76Y/E81A, which does not bind to PCh and a single mutant of CRP, E42Q, which binds to factor H (18, 46). A quadruple mutant of CRP (E-CRP-1), E42Q/F66A/T76Y/E81A, was constructed in which the F66A/T76Y/E81A mutations were introduced to abolish the PCh-binding and the E42Q mutation was added to confer the factor H-binding ability to mutated CRP. The ability to bind to PCh was abolished so that the observed effects could be attributed solely to the factor H-binding ability of E-CRP-1. While screening a library of CRP mutants for binding to factor H, we found that Y40F/E42Q CRP (E-CRP-2) bound to factor H more avidly than E42Q CRP did. That is why, E-CRP-2 was employed in this study, instead of E42Q CRP, as the molecule which binds to both PCh and factor H. The mechanism of interaction between various CRP mutants and immobilized factor H is currently being investigated in a separate project.

The data indicate that endogenous murine WT CRP also participated, along with E-CRP-1, in protecting mice against infection in our animal model. Both E-CRP-1 (cannot bind to PCh and hence unable to activate the complement system) and E-CRP-2 (can bind to PCh and hence able to activate the complement system) protected mice against late-stage infection. Since complement activation by CRP-complexes is necessary for protection (31), endogenous murine CRP must have participated along with E-CRP-1 in protecting mice against infection. Involvement of endogenous murine CRP in protection is also supported by the finding that the addition of human WT CRP to E-CRP-1 did not change the protective ability of E-CRP-1. The finding that the outcome of multiple injections of E-CRP-1 within 48 h of administering pneumococci was not different from a single injection at 12 h suggested that the effects of E-CRP-1 lasted for at least 48 h. Overall, the data indicate that CRP functions in two different conformations: in the WT conformation to bind to PCh and activate the complement system and in the altered conformation to bind to factor H to remove the inhibitory effect of factor H on complement activation. Experiments employing CRP-deficient mice are in progress to confirm the participation of endogenous murine CRP in E-CRP-1-mediated protection against late-stage infection.

As expected, E-CRP-1 acquired the ability of E42Q CRP to bind to factor H. Unexpectedly, CRP triple mutant, F66A/T76Y/E81A, which was not investigated before for factor H-binding (18), also bound to factor H, without the E42Q mutation (50). These results were not available till we generated and tested E-CRP-1 for binding to factor H. Since the expression of E-CRP-1 cDNA was higher than the expression of the triple mutant cDNA, we used E-CRP-1 in this study. Previously, we reported that CRP triple mutant, which does not bind to PCh, protected mice against infection and we interepreted the data to suggest that CRP protects mice against pneumococcal infection without binding to pneumococci. Recent findings that triple mutant CRP can also bind to factor H (50) and that complement activation is critical for protection (31) indicate that in previously published experiments employing CRP triple mutant (18, 30), endogenous murine CRP had also participated, along with triple mutant CRP, in protecting mice against infection.

**FIGURE 10:**
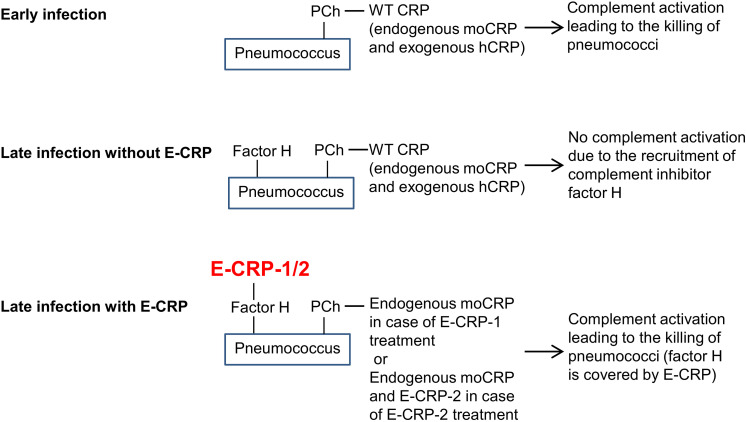
A proposed model for the mechanism of action of CRP in pneumococcal infection. moCRP, endogenous mouse WT CRP; hCRP, exogenously administered human WT CRP.

A model for the mechanism of action of E-CRP-1 and E-CRP-2 in protecting mice against infection is proposed (Figure 10). During early-stage infection, at a time when pneumococci have not recruited factor H yet on their surface to become complement-resistant, WT CRP is sufficient to decrease bacteremia. WT CRP (human or murine or both) would bind to PCh on pneumococci, activate the complement system, and reduce bacteremia. During late-stage infection, pneumococci recruit factor H and become resistant to WT CRP-activated complement-mediated killing. E-CRP-1 or E-CRP-2 would then bind to factor H on pneumococci, enabling complement activation by WT CRP/E-CRP-2-PCh complexes to proceed, resulting in the decrease in bacteremia. Thus, during late-stage infection, two different structural conformations of CRP are required for protection.

Based on the model for E-CRP-1 and E-CRP-2, we propose a possible mechanism of action of endogenous CRP in humans in pneumococcal infection. Pneumococci are usually harbored in the nasopharynx but can spread to lungs and bloodstream. From the blood, after evading the attack by the complement system, pneumococci can spread to multiple other organs causing septicemia (53–55). Pneumococci release toxic substances including H_2_O_2_, creating a localized inflammatory environment (56, 57). H_2_O_2_ has been found to modify the pentameric conformation of CRP, and H_2_O_2_-treated CRP binds to factor H (47). The presence of conformationally altered pentameric CRP has been shown to be one of the features of inflamed sites (40, 42, 58). The binding of conformationally altered CRP to factor H on complement-resistant pneumococci would result in converting pneumococci back to being complement-sensitive. WT CRP can then bind to PCh on pneumococci and activate the complement system to attack pneumococci. In this regard, CRP is similar to serum amyloid A (SAA) which is another acute phase protein in humans (59). SAA has recently been shown to exhibit pH-dependent antibacterial action against *Staphylococcus aureus*. Both CRP and SAA are produced not only by hepatocytes but also by a variety of cells in extrahepatic tissues (59–63). The expression of SAA is increased in abscesses of *S. aureus* cutaneous infected mice, and SAA then binds to bacterial cell surface and disrupts the cell membrane in acidic conditions (59). Similarly, it is possible that extrahepatically synthesized CRP may be responsible for the availability of conformationally altered CRP generated at sites of inflammation.

The finding that E-CRP-1 and E-CRP-2 bound to broth-grown pneumococci in the absence of Ca^2+^ suggests the involvement of a pneumococcal surface protein in the interaction between E-CRP-1/E-CRP-2 and pneumococci. Thus, the binding of E-CRP-1/E-CRP-2 to factor H may not be the only mechanism for protection against infection; as E-CRP-1/E-CRP-2 can interact directly with the surface virulence factors to eliminate their virulence. Since E-CRP-1 and E-CRP-2 bind to a variety of proteins immobilized on microtiter wells (data not shown), and not just to factor H, and because pneumococci also recruit other serum proteins to their surface, such as complement C1q, ficolins and complement inhibitor C4BP, our findings may be applicable to infections with a wide range of pneumococcal strains (64–71). The advantage of our strategy is that it is dependent on the recruited proteins and not on the serotype of pneumococci. We speculate that E-CRP could be therapeutically beneficial for infections with antibiotic-resistant pneumococcal strains, such as, strain 106 resistant to clindamycin, strain 109 resistant to clarithromycin, strain 999 resistant to penicillin, and others (72, 73). E-CRP could also be protective against infections with other bacterial species that use factor H to evade complement-mediated killing. For example, *Bordetella pertussis*, *Borrelia burgdorferi*, *Borrelia hermsii*, *Haemophilus influenzae*, *Neisseria gonorrhoeae*, *Neisseria meningitis*, *Pseudomonas aeruginosa*, and *Streptococcus suis*, are all known to recruit factor H (74–79). It is possible that CRP plays a general antibacterial role, as exemplified in studies that show CRP protecting mice against infection with *Salmonella typhimurium* also (80).

We conclude that CRP functions in two different structural conformations. Our data provide a proof of concept that the structure of CRP is subtly modified *in vivo* to execute full anti-pneumococcal activities. We hypothesize that in individuals in whom the conformation of CRP remains unchanged, perhaps due to inappropriate inflammatory conditions around CRP, CRP is not fully functional during infection. If this hypothesis is correct, then our findings provide a new strategy to treat pneumococcal infection by injecting exogenously prepared pre-modified CRP, such as E-CRP-1 and E-CRP-2. Pre-modified CRP could also be therapeutically beneficial for infections with antibiotic-resistant pneumococcal strains and for infections with other bacterial species that use host proteins to evade complement-mediated killing.

## Data Availability Statement

All datasets presented in this study are included in the article/Supplementary Material.

## Ethics Statement

The animal study was reviewed and approved by UCAC-ETSU.

## Author Contributions

DN, SS, and TG performed the experiments. AA conceived and designed the experiments. DN, SS, and AA analyzed the data. AA wrote the manuscript with input from DN. All authors contributed to the article and approved the submitted version.

## Conflict of Interest

The authors declare that the research was conducted in the absence of any commercial or financial relationships that could be construed as a potential conflict of interest.

## Abbreviations

CRP: C-reactive protein
E-CRP -1: CRP mutant E42Q/F66A/T76Y/E81A
E-CRP -2: CRP mutant Y40F/E42Q
PCh: phosphocholine
PEt: phosphoethanolamine
PnC: pneumococcal C-polysaccharide
Pn-broth: pneumococci cultured in broth
Pn-mice: pneumococci isolated from infected mice
WT: wild-type.
