# Supplementary material for: Treatment of Pneumococcal Infection by Using Engineered Human C-Reactive Protein in a Mouse Model
Source: Front Immunol. 2020 Oct 7;11:586669. doi: 10.3389/fimmu.2020.586669 (PMC7575696; doi:10.3389/fimmu.2020.586669)
Supplement: Supplementary file 1 [file Data_Sheet_1.PDF]

Supplementary Figure 1:
Nucleotide sequence of WT CRP (Page 1), E-CRP-1 (Page 2) and E-CRP-2 (Page 3),showing the codons for original and mutated amino acids.

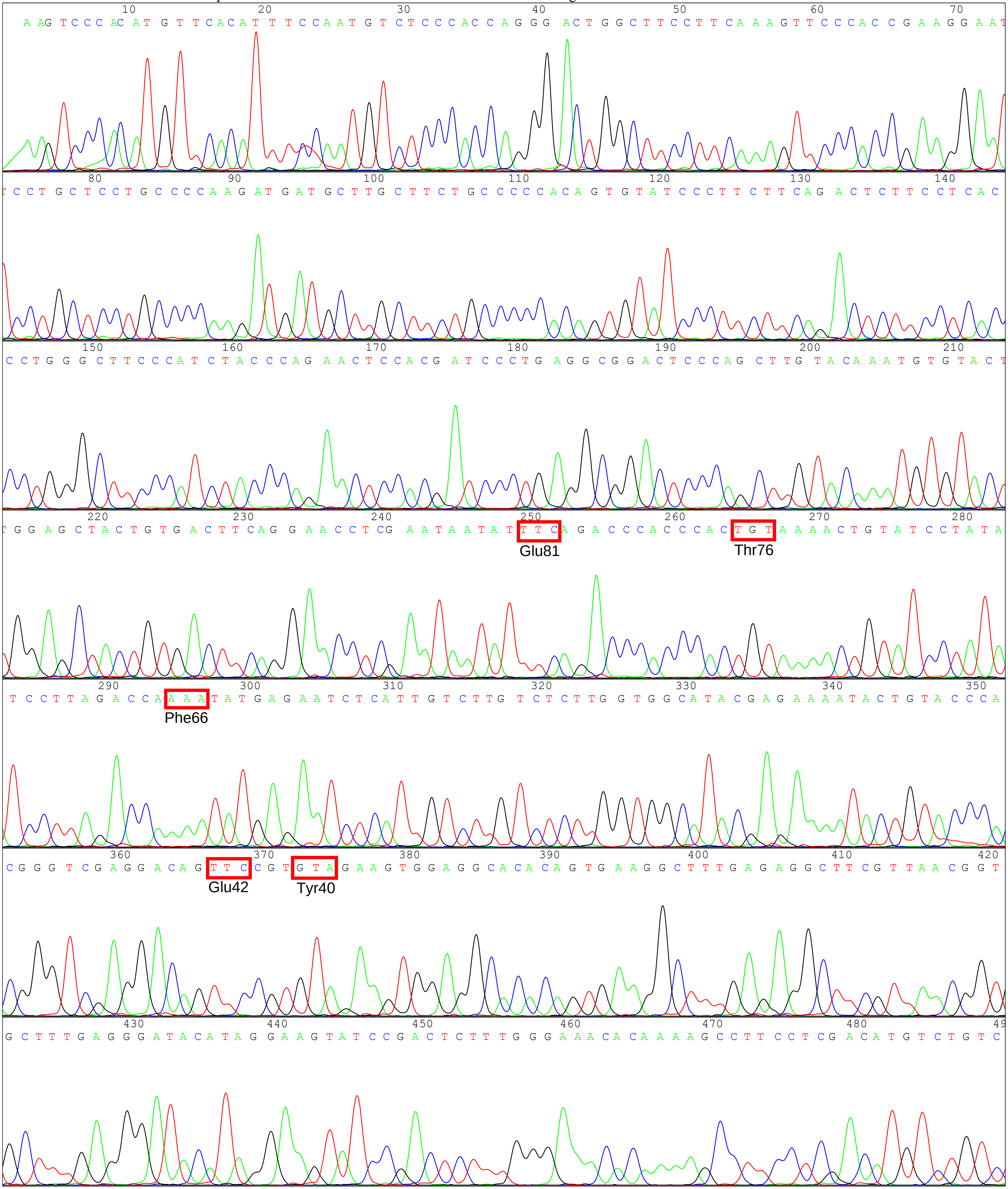
Glu81
Thr76
Phe66
Glu42
Tyr40

File: **E-CRP-1**.scf Sample: Primer-T173A-2 681 bases in 13275 scans Page 1 of 2

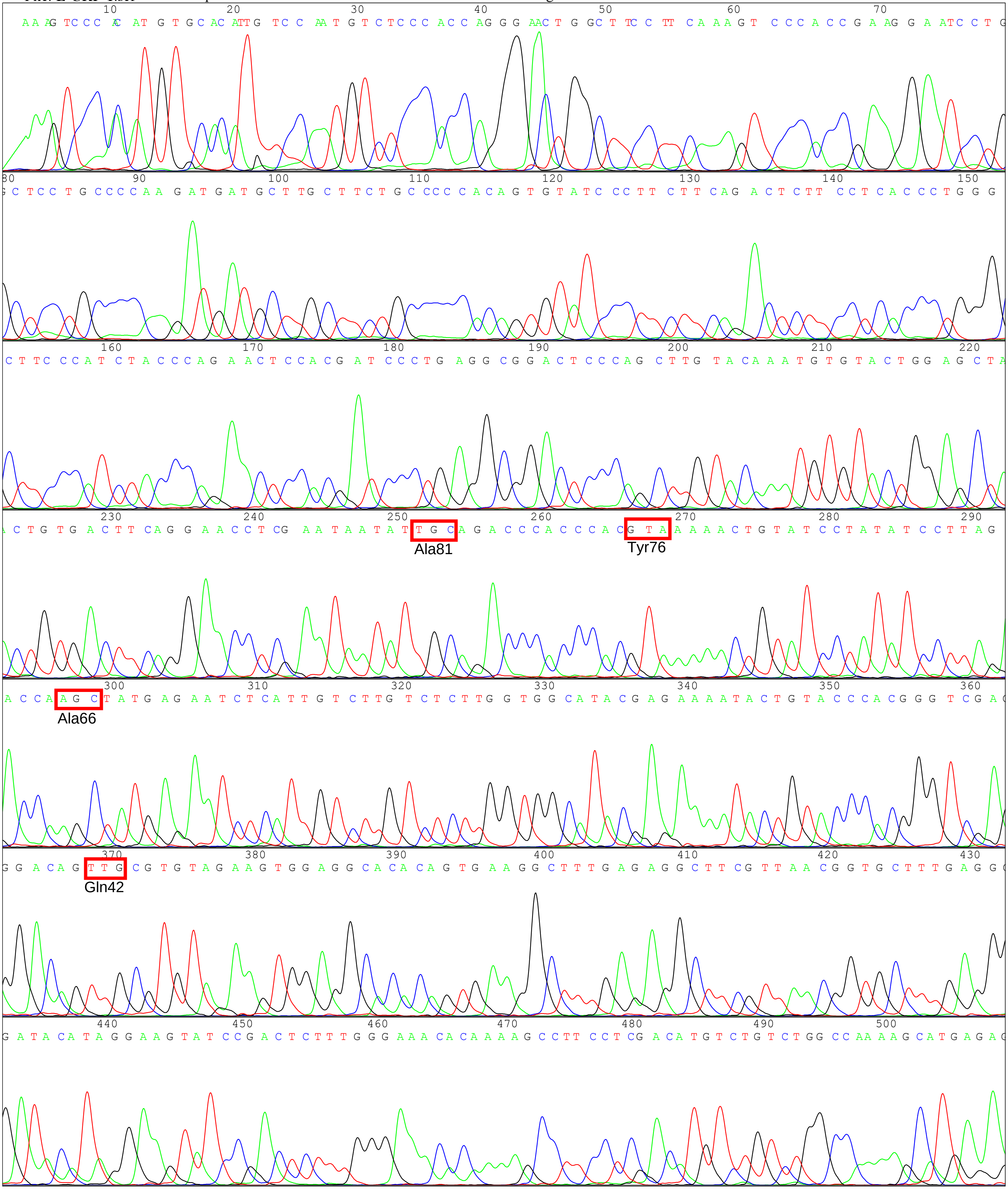

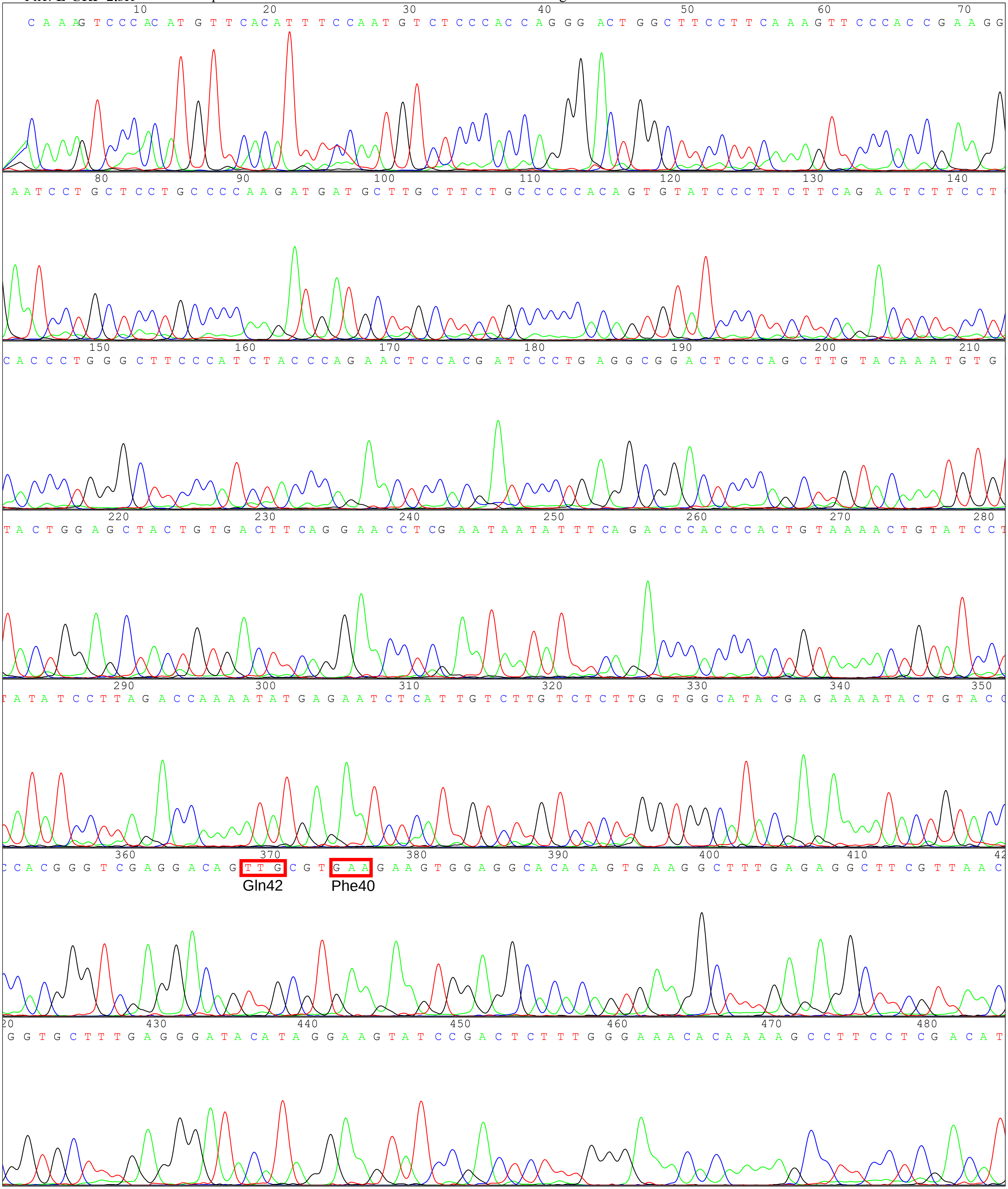
Gln42
Phe40
