# Supplementary material for: Treatment of Pneumococcal Infection by Using Engineered Human C-Reactive Protein in a Mouse Model
Source: Front Immunol. 2020 Oct 7;11:586669. doi: 10.3389/fimmu.2020.586669 (PMC7575696; doi:10.3389/fimmu.2020.586669)
Supplement: Supplementary file 2 [file Data_Sheet_2.PDF]

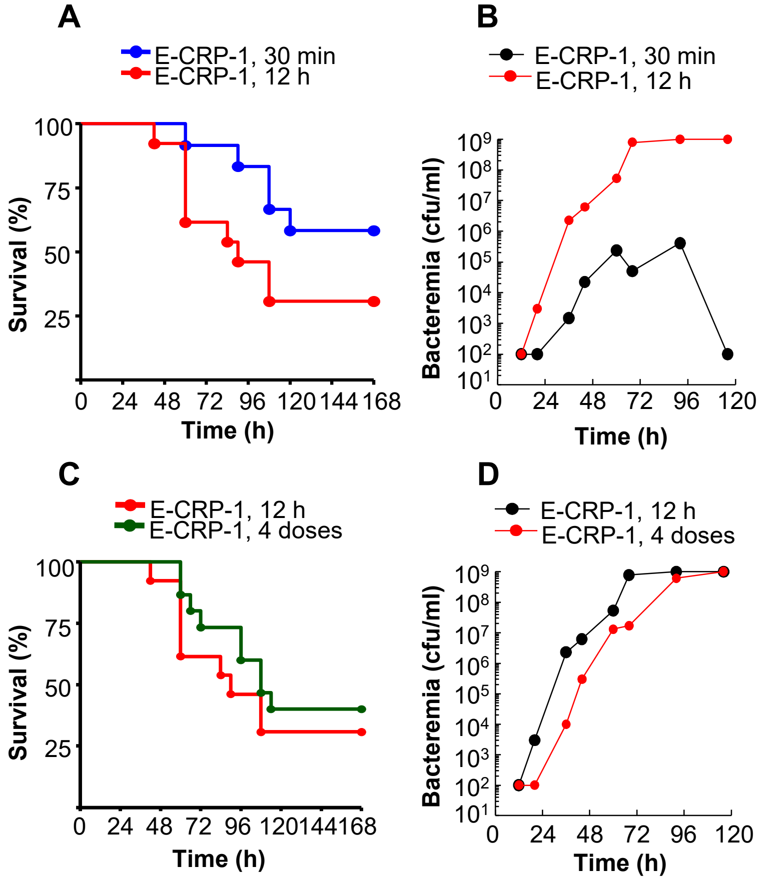

Supplemental Figure 2: E-CRP-1 was protective against late-stage infection regardless of the time of administration. The data shown in this figure are derived from **Figures 6-8.** (**A**) Survival curves. The *p* value for the difference between 30 min and 12 h survival curves was 0.09. (**B**) Median bacteremia in each group of mice shown in **A**. Statistical analysis for bacteremia was performed using the scatter plots shown in **Figures 6-7**. For 44-116 h, the differences in bacteremia between 30 min and 12 h groups were not statistically significant. (**C**) Survival curves. The *p* value for the difference between one dose and four doses survival curves was 0.41. (**D**) Median bacteremia in each group of mice shown in **C**. Statistical analysis for bacteremia was performed using the scatter plots shown in **Figures 7-8**. For 36-116 h, the differences in bacteremia between one dose and four doses groups were not statistically significant.
